# Azithromycin removal from water via adsorption on drinking water sludge-derived materials: Kinetics and isotherms studies

**DOI:** 10.1371/journal.pone.0316487

**Published:** 2025-01-09

**Authors:** Camilo C. Castro-Jiménez, Julio C. Saldarriaga-Molina, Edwin F. García, Ricardo A. Torres-Palma, Nancy Acelas

**Affiliations:** 1 Facultad de Ingeniería, Escuela Ambiental, Universidad de Antioquia UdeA, Medellín, Colombia; 2 Facultad de Ciencias Exactas y Naturales, Instituto de Química, Grupo de Investigación en Remediación Ambiental y Biocatálisis (GIRAB), Universidad de Antioquia UdeA, Medellín, Colombia; 3 Grupo de Investigación Materiales con Impacto (Mat&mpac) Universidad de Medellín, Medellín, Colombia; Jazan University, SAUDI ARABIA

## Abstract

In this study, we utilized drinking water treatment sludge (WTS) to produce adsorbents through the drying and calcination process. These adsorbents were then evaluated for their ability to remove azithromycin (AZT) from aqueous solutions. The L-500 adsorbent, derived from the calcination (at 500°C) of WTS generated under conditions of low turbidity in the drinking water treatment plant, presented an increase in the specific surface area from 70.745 to 95.471 m^2^ g^-1^ and in the total pore volume from 0.154 to 0.211 cm^3^ g^-1^, which resulted in a significant AZT removal efficiency of 65% in distilled water after 60 min of treatment. In synthetic wastewater, the rate of AZT removal increased to 80%, in comparison, in a real effluent of a municipal wastewater treatment plant, an AZT removal of 56% was obtained. Kinetic studies revealed that the experimental data followed the pseudo-second-order model (R^2^: 0.993–0.999, APE: 0.07–1.30%, and Δq: 0.10–2.14%) suggesting that chemisorption is the limiting step in the adsorption using L-500. This finding aligns with FTIR analysis, which indicates that adsorption mechanisms involve π-π stacking, hydrogen bonding, and electrostatic interactions. The equilibrium data were analyzed using the nonlinear Langmuir, Freundlich, and Langmuir-Freundlich isotherms. The Langmuir-Freundlich model presented the best fitting (R^2^: 0.93, APE: 2.22%, and Δq: 0.06%) revealing numerous interactions and adsorption energies between AZT and L-500. This adsorbent showed a reduction of 19% in its AZT removal after four consecutive reuse cycles. In line with the circular economy principles, our study presents an interesting prospect for the reuse and valorization of WTS. This approach not only offers an effective adsorbent for AZT removal from water but also represents a significant step forward in advancing sustainable water treatment solutions within the framework of the circular economy.

## Introduction

The United Nations strongly emphasizes the importance of promoting healthy lives for sustainable development. As a result, one of the key targets of Sustainable Development Goal -SDG- 3 (Good health and well-being) is to reduce the deaths and illnesses caused by water contamination [1]. In this context, antibiotics, classified as emerging contaminants (EC), have been frequently found in natural water sources, prompting widespread concern [2]. These pharmaceuticals are of particular concern due to the potential health and environmental risks associated with antimicrobial resistance [3–5].

Among the antibiotics, azithromycin (AZT) has been recognized as a priority substance on the second European Union watch list [6]. AZT belongs to the macrolide class, a group of antibiotics frequently detected in effluents, sewage sludge, sediments, soils, and food [7]. AZT is a broad-spectrum antibiotic used for various respiratory diseases, including COVID-19 [8–10], also AZT plays a crucial role in the medical field as a widely used antibiotic for treating various bacterial infections [11]. During the COVID-19 pandemic, pharmaceuticals like AZT were extensively used in treatment efforts [12]. This heightened usage resulted in increased excretion of these antibiotics into the environment via human waste [13].

Pharmacokinetic studies of AZT indicate that the body does not fully absorb it and that it can be excreted in its primary form into wastewater [12]. Additionally, no human metabolites or transformation products of this antibiotic have been identified [14]. Effluents from antibiotic manufacturing plants have been identified as another considerable source and pathway for the release of antibiotics like AZT, into aquatic environments [15].

When azithromycin enters aquatic environments, it can persist for extended periods, potentially affecting aquatic organisms and ecosystems. Its presence may also contribute to the emergence of antibiotic-resistant bacteria, posing threats to both human and animal health [16]. Moreover, the slow metabolism of AZT suggests that it may be poorly degraded in sewage treatment plants [17].

AZT has been detected in surface water sources in countries such as Spain (3 ng L^-1^) and China (4.3 ng L^-1^ to 935 μg L^-1^), as well as in surface water sediments (24 ng L^-1^) and groundwater (257 ng L^-1^) in Spain [18]. It has also been reported in raw and treated domestic wastewater in countries like the United States (raw: 18.3 μg L^-1^, treated: 3.25 μg L^-1^) and Colombia (raw: 5.24–7.00 μg L^-1^, treated: 3.02–4.66 μg L^-1^) [19]. In raw hospital wastewater, AZT concentrations range from 6.93 to 26.1 μg L^-1^ in Colombia [19] and 163 μg L^-1^ in Turkey [18]. The post-pandemic surge in AZT concentration in surface water, wastewater, and treated wastewater [18] and the ineffective conventional wastewater treatment methods for removing AZT [19] underline the urgency of developing alternative treatment approaches to effectively eliminate it from water [20].

At present, several treatments have been evaluated for removing AZT from aquatic systems, including adsorption [21–28], membrane filtration [29], advanced oxidation processes -AOPs- [30–33], and biological systems [34–38]. However, traditional methods like biological treatments face challenges due to their low efficiency and slow antibiotic decomposition rates [39]. Adsorption is considered as a highly effective, convenient, simple design, and cost-efficient method for eliminating pollutants from water without generating undesirable byproducts which can be produced in oxidation processes [2,8].

Adsorption is a surface process in which contaminants are transferred from a fluid bulk to the adsorbent surface through physical forces or chemical bonds [40]. Various adsorbents have been studied for the removal of AZT from water, including biochar, lignite, polyamide nanofibers [21], PAC (Powdered Activated Carbon) combined with Fe/Ag/Zn nanocomposites [22], activated porous carbon derived from the water fern *Azolla filiculoides* [23], mesoporous silica materials synthesized via the hydrothermal method [24], organoclays [25], natural clinoptilolite modified with surfactants [26], raw and modified nano-diatomites [27], and powdered zeolites [28].

Producing adsorbents from waste can enhance the process’s economic viability and exemplify waste valorization, aligning with the principles of the circular economy [41]. Among the waste materials that have garnered special interest for producing adsorbents are water treatment sludges (WTS). This waste is generated worldwide as a byproduct of drinking water treatment [42]. An average of 100,000 tons of WTS is estimated to be produced globally daily, which may triple in the coming decades [43]. Numerous studies have assessed adsorbents derived from WTS, particularly for removing metals, metalloids, phosphorus species, fluorides, and dyes across various water matrices [44].

However, water treatment sludges (WTS) have received relatively little exploration as a source material for antibiotic adsorption. To the best of our knowledge, the studies by He et al. [2], Punamiya et al. [45], and Saman [46] are the only ones to date that have reported on the adsorption of antibiotics from the tetracycline group by adsorbents made from WTS. Their findings highlight the need for further studies to explore the application of these adsorbents in real systems and to evaluated the effects of the adsorption process in complex matrices. To our knowledge, no studies have been reported on using adsorbents derived from WTS to remove the antibiotic AZT from water or complex matrices, such as simulated and real wastewater treatment plant effluents.

This study investigates the adsorption of the antibiotic AZT, using adsorbents produced by simple methods such as drying and calcinating of water treatment sludge (WTS), an easily obtained waste generated worldwide by drinking water treatment systems. Initially, three types of WTS were evaluated, each generated from raw water of varying qualities in the drinking water treatment plant. The key factors influencing AZT adsorption with the adsorbent that exhibited the most promising results were examined, including adsorbent dose, adsorption pH, the size of adsorbent particles and reuse. Furthermore, the experimental data were fitted to nonlinear adsorption kinetics models and adsorption isotherms for a more comprehensive understanding of the results. Additionally, AZT adsorption was studied in simulated and real municipal wastewater treatment plant effluents to assess the matrix impact. This study’s innovative use of WTS, combined with the detailed analysis of adsorption kinetics and isotherms, practical applicability in different water matrices, and alignment with circular economy principles, underscores the novelty and significance of the research.

## Materials and methods

### WTS sampling

Water treatment sludge samples (WTS) were collected from the settlers of a conventional drinking water treatment plant (DWTP) in Colombia. This DWTP employs aluminum sulfate as a coagulant for treating surface water. Three WTS samples were obtained, each corresponding to different turbidity levels in the source water and varying coagulant doses, as detailed in Table 1. WTS types were categorized based on a statistical analysis of raw water turbidity data from the DWTP spanning a 5 years period.

**Table 1:** WTS categorization and operational parameters of the DWTP.

WTS type	Notation	Operational parameters of the DWTP		
		Raw water turbidity (NTU)	Coagulant dose (mg alum L^-1^)	WTS frequency ^a^ (%)
Low turbidity WTS	L-WTS	< 5	4	53
Medium turbidity WTS	M-WTS	5–10	6	32
High turbidity WTS	H-WTS	10–30	8	15

^a^ WTS frequency represents the percentage of months during the observation period in which the 90th percentile of raw water turbidity measurements falls within the range defined for the specific sludge type.

AZT powder was obtained from Zhejiang Guobang Pharmaceutical Co., Ltd., hydrochloric acid (HCl), sulfuric acid (H_2_SO_4_), sodium hydroxide (NaOH), sodium chloride (NaCl), ethanol (C_2_H_6_O) and methanol (CH₃OH) were obtained from Merck S.A. (Germany). In this study, distilled water was used for aqueous solutions.

### WTS adsorbents preparation

**Fig 1:**
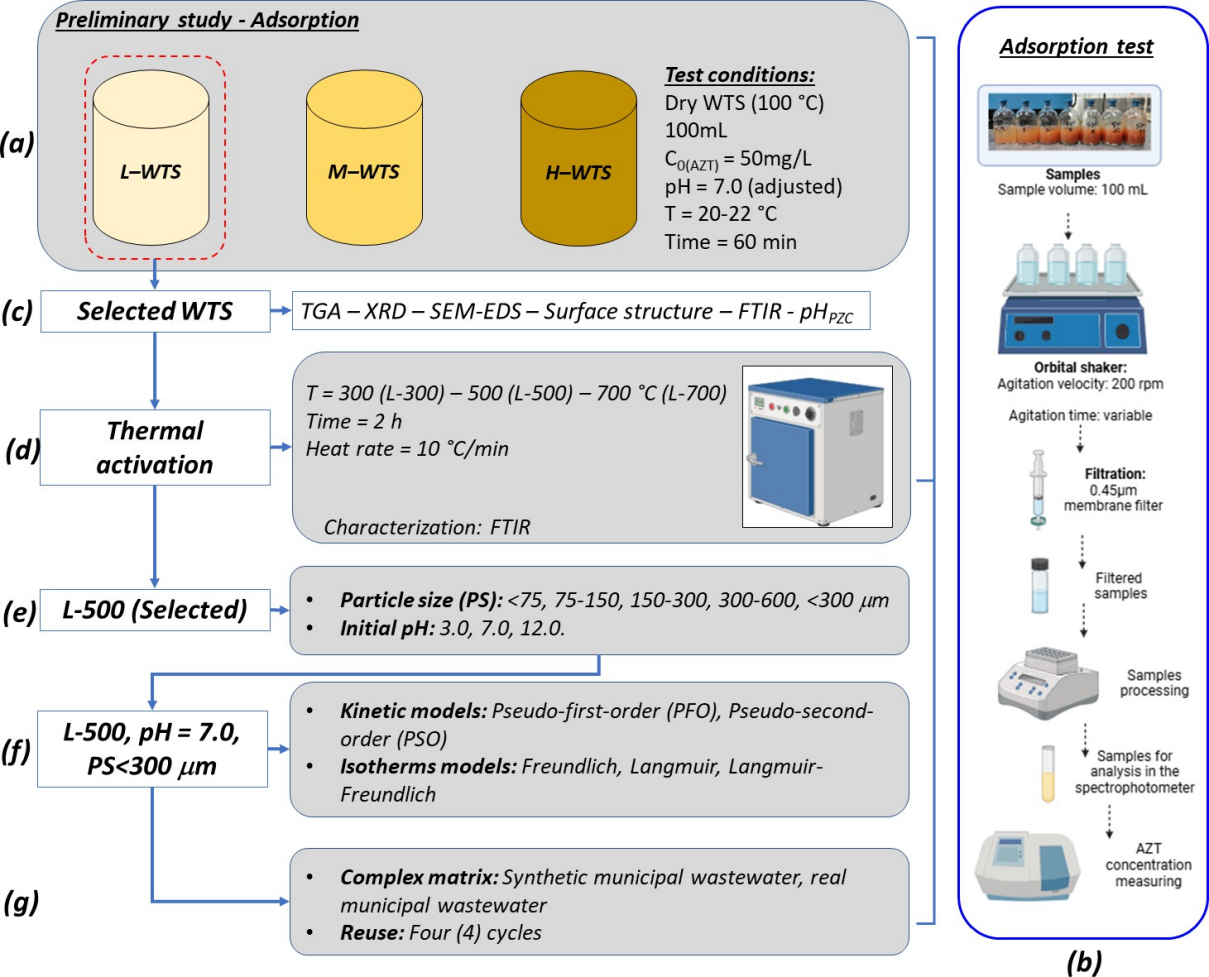
Experimental design sketch of the optimization study.

Each WTS was initially dried in an oven at 100°C until a constant weight was achieved. Subsequently, they were ground and sieved (< 300 μm) to obtain dry WTS samples designated as L-100, M-100, and H-100. To assess the impact of calcination temperature, adsorbents were further prepared at 300°C (L-300), 500°C (L-500), and 700°C (L-700) using L-WTS. These calcinations were conducted in a furnace under an oxygen atmosphere for 2 h, with a heating rate of 10°C per minute. Finally, each calcined WTS sample was ground and sieved (< 300 μm) (Fig 1).

### Characterization of WTS adsorbents

The specific surface area and pore structure of the absorbents were determined by the Brunauer, Emmett, and Teller (BET) method [47] with N_2_ adsorption at 77 K using an accelerated surface area and porosimeter system (Micromeritics, ASAP 2020 Plus). The surface chemical composition and morphology of the L-WTS were characterized by scanning electron microscopy (SEM, JEOL JSM 6490 LV) equipped with an Oxford energy-dispersive spectroscopy (EDS) system. Fourier transform infrared spectroscopy (FTIR, Spectrum-two, Perkin Elmer with UATR) was used to analyze the active functional groups of the samples for a range within 4000–450 cm^−1^. The zero-charge point pH (pH_PZC_) was calculated using the solids addition method [48]. The minerals in L-WTS were determined using an Empyrean 2012 X-ray diffractometer (Malvern-PANalytical) with copper [Cu, Kα = 0.15406 nm (1.5406 Å)] as the radiation source. Quantification was performed with the software HighScore Plus, using the Rietveld method and the ICSD FIZ Karlsruhe 2012–1 database.

### Adsorption experiments

Initially, AZT removal was evaluated using adsorbents derived from three distinct types of WTS. Preliminary adsorption experiments were conducted with varying doses of the adsorbent. Each experiment involved 100 mL of a 50 mg L^-1^ (C_0_) azithromycin (AZT) solution, prepared in distilled water at a pH of approximately 7.0 (adjusted) and performed at room temperature (20–22°C) for 60 minutes (Fig 1A). The S1 Text details the AZT solution preparation procedure. The bottles were agitated at 200 rpm using an orbital shaker throughout the adsorption process. Samples were extracted at specific time intervals (t) and immediately filtered through a 0.45μm filter membrane (Fig 1B). The concentration of AZT (C_t_) in the filtered samples was determined using a colorimetric method, as summarized in S2 Text [27,49–51]. The AZT removal was calculated according to Eq (1):

$$AZT\;removal\;(\%)=\left(\frac{C_{0}-C_{t}}{C_{0}}\right)\times 100$$ (1)

Where *C*_*0*_ (mg L^-1^) and *C*_*t*_ (mg L^-1^) represent the AZT concentrations at time 0 and *t*, respectively.

Considering the results of AZT removal and the prevalence of a specific type of WTS generated in the DWTP (as shown in Table 1), we chose L-WTS to investigate the impact of adsorbent preparation temperature on AZT removal (Fig 1C). Subsequently, using L-WTS calcined at 500°C (L-500) (Fig 1D), we explored the influence of pH (ranging from 3 to 12) and adsorbent particle size (<75, 75–150, 150–300, 300–600, and <300 μm) on AZT removal (Fig 1E). The adsorption capacity (q_t_) was determined using Eq (2).


$$q_{t}\left(mg\cdot g^{-1}\right)=\left(\frac{C_{0}-C_{t}}{m}\right)\times V$$ (2)

Where *m* (g) represents the mass of the adsorbent, *V* (L) stands for the volume of the AZT solution, *C*_*0*_ (mg L^-1^) and Ct (mg L^-1^) have been previously defined.

### Adsorption kinetic and isotherms models

At the optimal adsorption pH, the kinetics and isotherms of the adsorption process of AZT onto L-500 were evaluated (Fig 1F). In their nonlinear forms, the pseudo-first-order (PFO) and pseudo-second-order (PSO) models were employed to fit the kinetic experimental data. This choice was made because linearizing these models can introduce uncertainties and propagate errors, leading to inaccurate estimations of the model parameters [52,53]. Eqs (3) and (4) represent the kinetic models:

$$\underline{\text{Pseudo-first}\;\text{order:}}\;q_{t}=q_{e}\left(1-e^{-k_{1}t}\right)$$ (3)

$$\underline{\text{Pseudo-second}\;\text{order:}}\;q_{t}=\frac{q_{e}^{2}k_{2}t}{1+q_{e}k_{2}t}$$ (4)

Where *k*_*1*_ (min^-1^) is the pseudo-first-order adsorption rate constant; *q*_*t*_ (mg g^-1^) and *q*_*e*_ (mg g^-1^) are the adsorption capacities at equilibrium and time *t* (min), respectively; *k*_*2*_ (g mg^-1^ min^-1^) is the pseudo-second-order adsorption rate constant. The constants of the kinetic models were calculated using the least squares model derived from the Rosenbrock-Newton optimization algorithm by Statistica software. Three isothermal models, Freundlich [54], Langmuir [55], and Langmuir–Freundlich [56–58] were applied to analyze the AZT adsorption on L-500 at the equilibrium. The mathematical models are presented in Table 2 (Eqs (5)–(8)). The coefficient correlation (R^2^), the average percentage error (APE) (S3 Text) [59], and the normalized standard deviation (Δq) (S3 Text) were used to validate the kinetics and isothermal models [60].

**Table 2:** Isotherm models.

Isotherm	Mathematical model	Equations
**Freundlich**	$q_{e}=K_{F}C_{e}^{1/n}$	(5)
**Langmuir**	$q_{e}=\frac{q_{m}K_{L}C_{e}}{1+K_{L}C_{e}}$ $R_{L}=\frac{1}{1+K_{L}C_{0}}$	(6) (7)
**Langmuir–Freundlich**	$q_{e}=\frac{q_{m}{\left(K_{a}C_{e}\right)}^{n_{LF}}}{{\left(K_{a}C_{e}\right)}^{n_{LF}}+1}$	(8)

Where q_e_ (mol kg^-1^) and C_e_ (mol L^-1^) are the AZT concentration and the adsorption capacity at equilibrium, respectively; K_F_ (mol kg^-1^) and n are Freundlich constants representing the multilayer adsorption capacity and the adsorption intensity, respectively; q_m_ (mol kg^-1^) is the maximum adsorption capacity, K_L_ (L mol^-1^) is the Langmuir constant, R_L_ is separation factor, and C_0_ (mol L^-1^) is the initial AZT concentration; K_a_ (L mol^-1^) is the adsorption affinity constant and n_LF_ is the index of heterogeneity. R_L_ serves to predict whether the adsorption process is favorable (R_L_ < 1), unfavorable (R_L_ > 1), irreversible (R_L_ = 0), or linear (R_L_ = 1) [61].

### Reusability tests and complex matrix evaluation

The performance of the L-500 material was assessed over three reuse cycles under optimal conditions (Fig 1G). Batch systems were exposed to an AZT solution (100 mg L⁻¹) for 60 minutes with agitation at 200 rpm using an orbital shaker (Fig 1B). After the adsorption phase, the material was vacuum-filtered and treated with ultrasound using a Digital Pro (model: PS-30AL) for 30 minutes at 40 kHz. This treatment was performed in a mixture of 33.3 mL methanol (CH₃OH) and 6.6 mL of 3% sodium hydroxide (NaOH) solution. The material was then washed under vacuum with deionized water, dried at 100°C for 24 hours, and prepared for the next adsorption cycle [62].

Additionally, the AZT adsorption within a complex matrix was assessed by introducing 5 g of L-500 into a 100 mL synthetic wastewater solution spiked with AZT at a concentration of 100 mg L^-1^. This wastewater solution was prepared using the methods described by Paredes-Laverde et al. [63] and OECD [64], simulating the effluent of a municipal wastewater treatment plant (as detailed in S1 Table). Similarly, the adsorption of AZT was evaluated in a real effluent from a domestic wastewater treatment plant, using the same procedure as applied to the synthetic wastewater matrix (Fig 1G). All experiments were conducted at least by duplicate.

## Results and discussion

### Preliminary results

**Fig 2:**
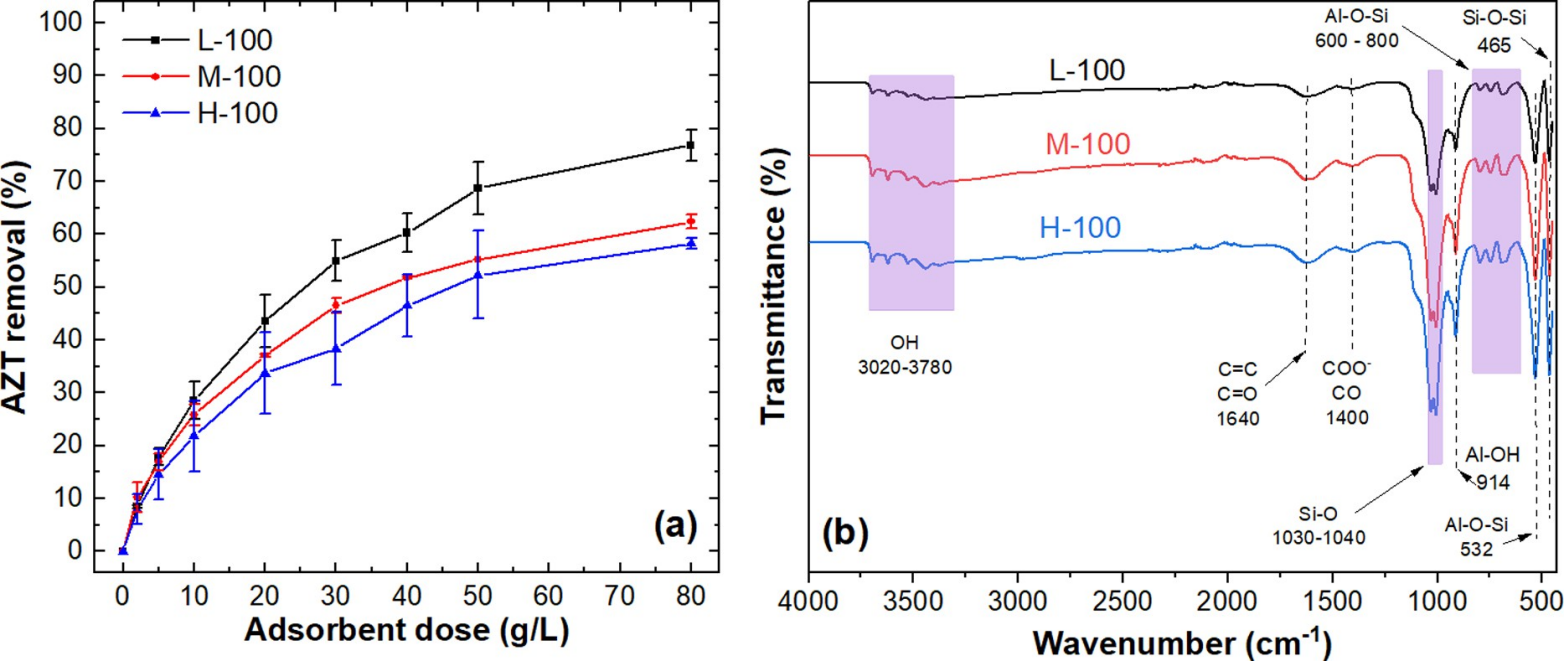
(a) Effect of dried WTS doses on AZT removal, (b) FTIR spectra of L-100, M-100 and H-100. Experimental conditions: C _0_: (a) 50 mg AZT L^-1^, contact time (t): 60 min, pH: 7.0, particle size (PS): <300 μm, temperature (T): 20–22°C.

Fig 2A shows the AZT removal achieved with the three types of dry WTS by using several adsorbent doses (from 2.5 to 80 g L^-1^). It can be noted that similar AZT removal percentages were obtained for the dry WTS from sources with medium (M-100) and high turbidity (H-100), peaking at around 60% with an adsorbent dose of 80 g L^-1^. The highest AZT removal (~77% at the higher dose) was achieved with the WTS generated under conditions of low turbidity in the raw water (L-100). The AZT removals obtained with the three WTSs may be associated with the existence of the silica network functional groups observed from 465 to 1150 cm^-1^ in Fig 2B [65]. These functional groups have been reported as active sites of interaction with AZT through to the electrostatic interaction and H-bonding [28]. As the L-WTS had the highest AZT removal and is the most frequently produced sludge type at the DWTP, this WTS was selected for further investigation. The characterization of the L-WTS is presented in the following section.

### L-WTS characterization

**Fig 3:**
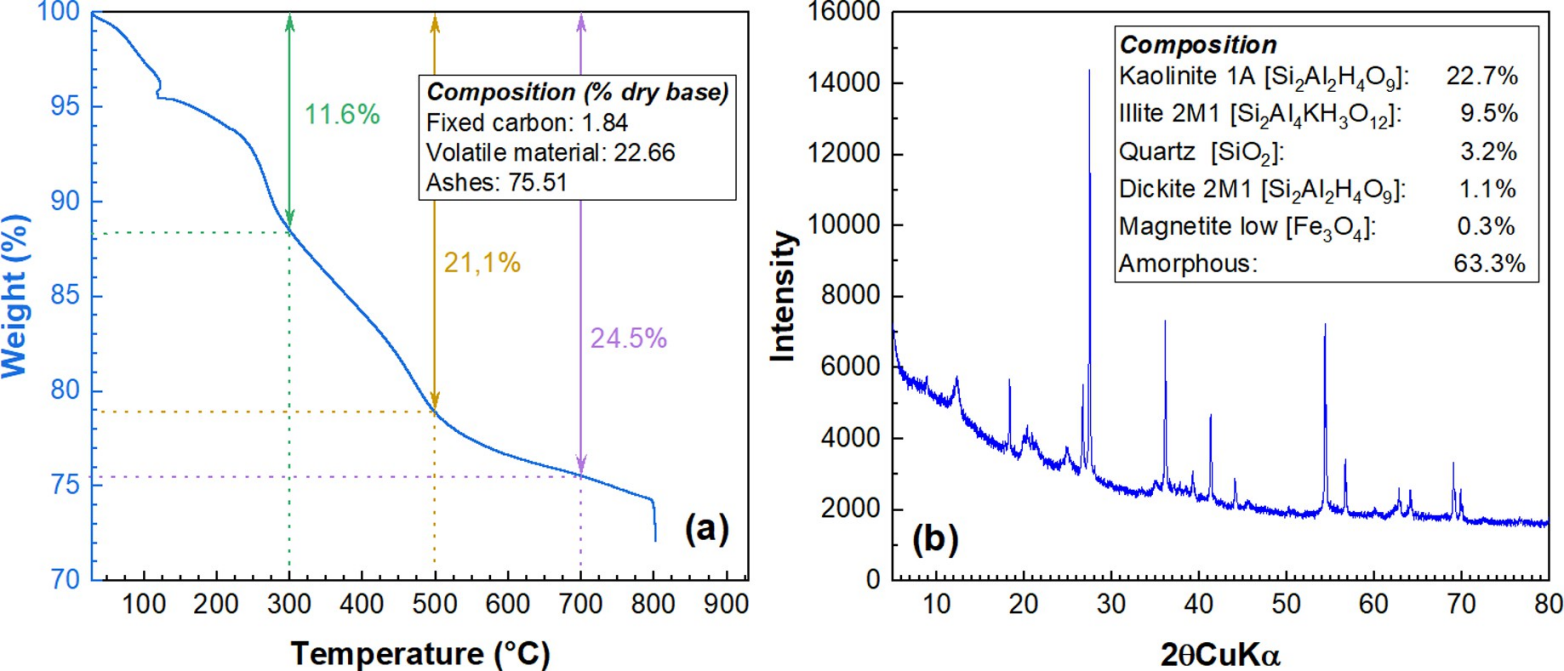
(a) TGA analysis of L-WTS, (b) XRD pattern of L-WTS.

Fig 3A presents the thermogravimetric analysis of L-WTS. The analysis reveals that L-WTS undergoes a significant mass change up to approximately 200°C, attributed to the evaporation and hydration-induced loss of water, as Martins et al., [66] reported. Subsequently, between 200 and 600°C, a mass loss is attributed to the degradation of natural organic matter -NOM- [67]. Furthermore, the results from the X-ray diffraction pattern (Fig 3B) suggest that L-WTS is primarily amorphous (comprising 63.3%), with the few crystalline structures present being aluminum-silicate clays (kaolinite 1A, illite 2M1, and dickite 2M1), quartz, and low-level magnetite. These structures originate from particles in the raw water that are removed during water treatment.

**Fig 4:**
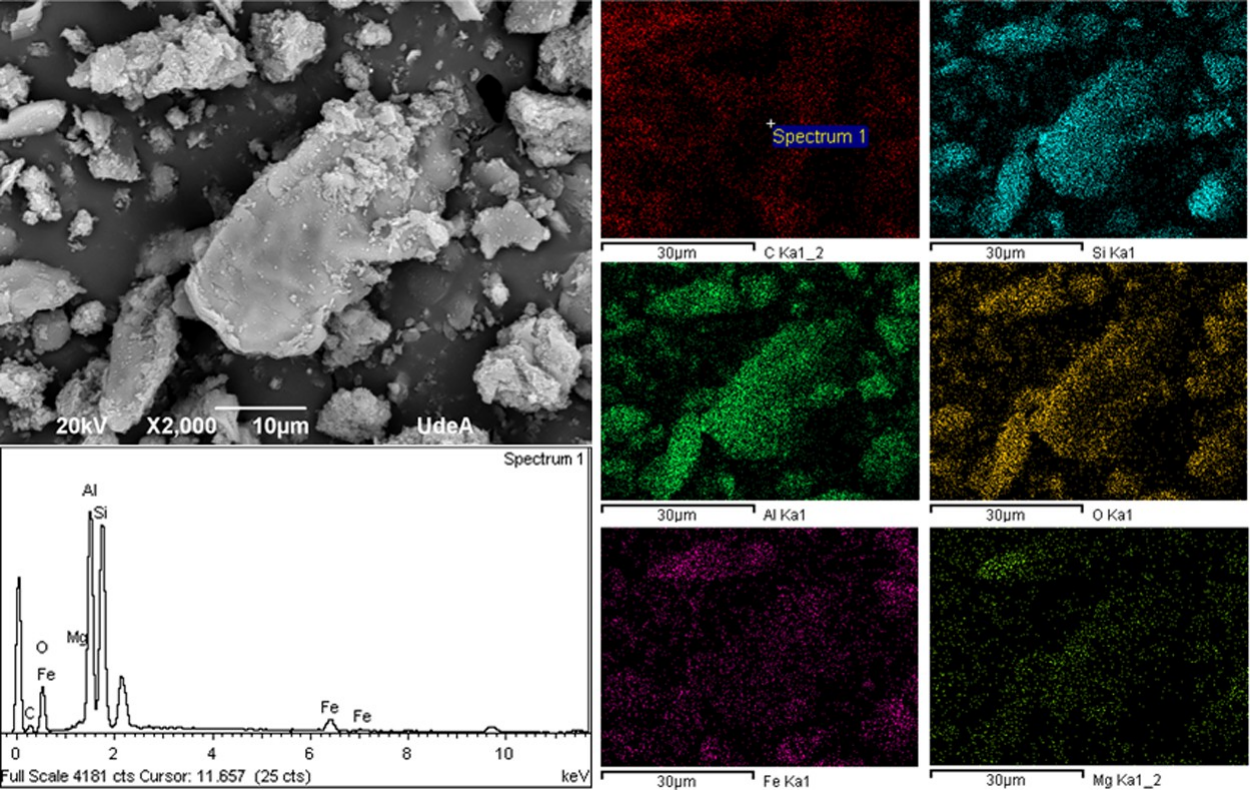
SEM images and the EDS spectra of the L-WTS.

The surface chemical composition and morphology of L-WTS are depicted in Fig 4. Notably, particles with various sizes, irregular shapes, and rough texture can be observed. According to the results from EDS spectra, the surface of L-WTS is primarily composed of Si, Al, C, O, and Fe, commonly found in sludges from DWTPs [44].

### Effect of thermal activation of L-WTS on AZT removal

**Fig 5:**
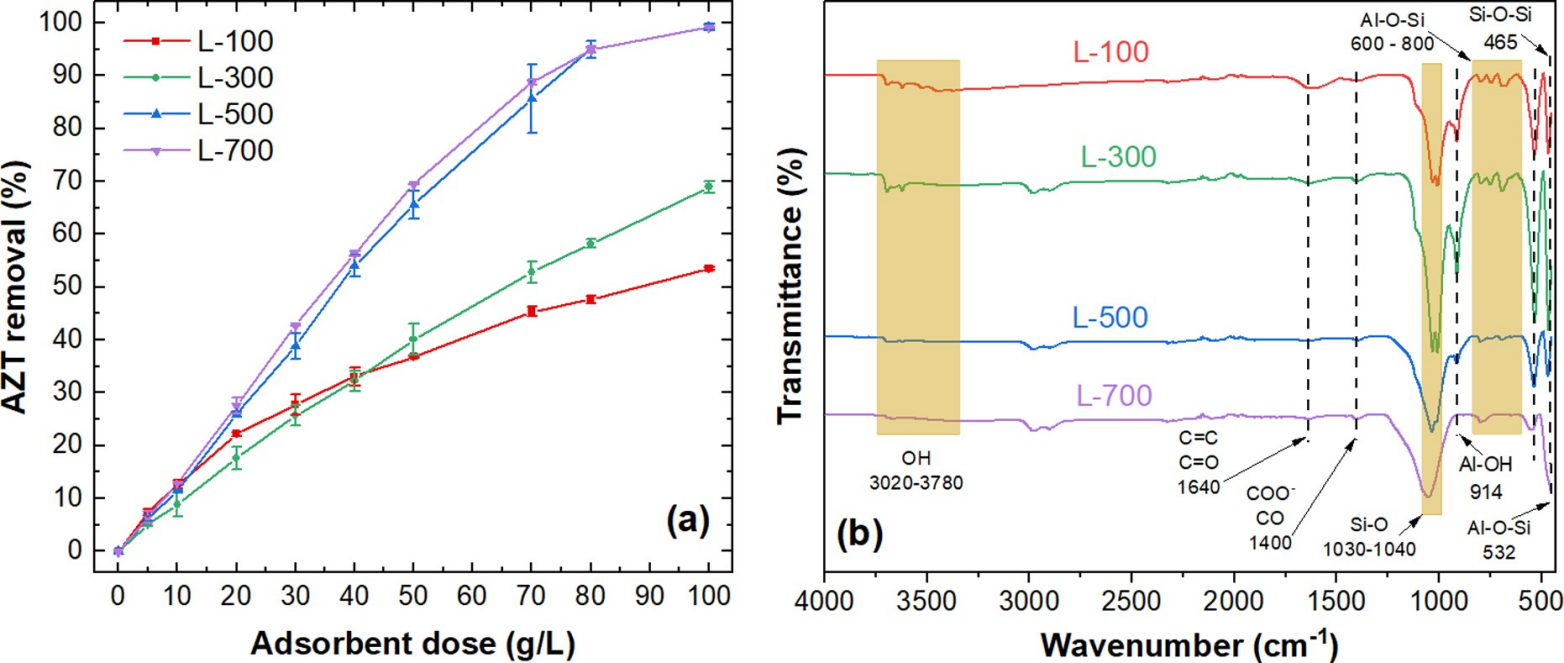
(a) Effect of thermal activation of L-WTS on AZT removal by varying the adsorbent dosage, (b) FTIR spectra of L-100, L-300, L-500, and L-700. Experimental conditions: C _0_: (a) 100 mg AZT L^-1^, contact time (t): 60 min, pH: 7.0, particle size (PS): <300 μm, temperature (T): 20–22°C.

The impact of the thermal activation of L-WTS on AZT adsorption by varying the adsorbent dosage was examined, as depicted in Fig 5A. Notably, adsorbents prepared at 500°C (L-500) and 700°C (L-700) exhibited the highest AZT removal percentages across various doses. Using these adsorbents, nearly complete removal of AZT (>99%) was achieved in 100 min with a dose of 100 g L^-1^. Calcination has been shown to enhance AZT adsorption, as reported by Saman et al. [46], who observed similar effects in the removal of cationic and anionic dyes as well as the antibiotics tetracycline and oxytetracycline. Considering its superior AZT removal efficiency and its lower energy for the adsorbent preparation compared to L-700, L-500 was selected for further adsorption experiments.

To elucidate the enhancement in AZT adsorption by L-500, the surface structure and functional groups of L-500 and L-100 were examined. Table 3 and S1 Fig exhibit the specific surface area and pore structures of the L-100 and L-500. Calcinating L-100 at 500°C resulted in a notable increase in its specific surface area (S_BET_) from 70.745 m^2^ g^-1^ to 95.471 m^2^ g^-1^. Similarly, it led to a 37% increase in the total pore volume of L-100, rising from 0.154 cm^3^ g^-1^ to 0.211 cm^3^ g^-1^. Both materials displayed a predominant pore size distribution centered around 3.5 nm, with an average pore diameter of around 9.0 nm, indicating a mesoporous structure (S1 Fig, inset). These findings align with previous reports on the thermal modification of WTS at similar temperatures (475–550°C) and highlight that the calcination of WTS can enhance active sites and pore diffusion capacity for pollutant adsorption [44,68].

**Table 3:** Surface structure of L-100 and L-500.

WTS type	Specific surface area—S_BET_ (m^2^ g^-1^)	Micropore area (m^2^ g^-1^)	External surface area (m^2^ g^-1^)	Total pore volume (cm^3^ g^-1^)	Micropore volume (cm^3^ g^-1^)	Average d_pore_ (nm)
**L-100**	70.745	17.911	52.834	0.154	0.007	8.71
**L-500**	95.471	18.701	76.770	0.211	0.008	8.84

The functional groups present in L-100, L-300, L500, and L-700 characterized by ATR-FTIR are depicted in Fig 5B. The band observed from 3020 to 3780 cm^-1^ in L-100 and L-300 corresponds to the OH stretching vibrations of sorbed water, mineral hydroxide phases, and organic hydroxide [68,69]. The decrease in intensity of this band in WTS observed after calcination at 500°C (L-500) and 700°C (L-700) can be attributed to increased decomposition of NOM and mineral dehydration [67]. NOM in the L-100 is associated with bands around 1640 cm^−1^ (C = C, C = O) and 1400 cm^-1^ (COO-, C-O). The presence of NOM in L-100 and L-300 may limit its adsorption capacity by competition with the target pollutant. The thermal treatment at 500°C and 700°C produces a loss of NOM, and the peaks corresponding to the bands 1640 cm^−1^ and 1400 cm^−1^ practically disappear in L-500 and L-700, which can enhance its adsorption capacity [67,69]. Other bands attributed to the silica network fall within the range of 465–1150 cm^-1^ [65]. These bands are related to the structures of the quartz and the clays identified in XRD analysis. The peaks at approximately 1030 cm^−1^, 532 cm^−1^, and 465 cm^−1^ are associated with Si–O vibrations, as indicated by Shamaki et al. [68]. These peaks persisted in L-500 and L-700, confirming the stability of the SiO_2_ phase following calcination. The band at 914 cm^−1^ is associated with the elongation of Al-OH bonds [66]. As demonstrated by the results of L-500 and L-700 samples, calcination reduces this band due to the dehydroxylation of clay minerals presented in L-100 and reported on XRD analysis [68]. The results of surface characterization for L-100 and L-500 suggest that the significant improvement in AZT removal observed with L-500 is primarily linked to enhancements in its surface structure (an increase in surface area and pore volume) and the NOM removal resulting from the thermal treatment at 500°C. As the AZM molecule is quite large, its adsorption onto L-100 and L-500 is supposed to primarily involve external surface interactions [28]. This is due to the limited ability of the molecule to access the adsorbent’s micropores. Also, these micropores only represent a small fraction of the total pore volume and S_BET_ for both adsorbents (refer to Table 3).

### Effect of particle size on AZT adsorption

**Fig 6:**
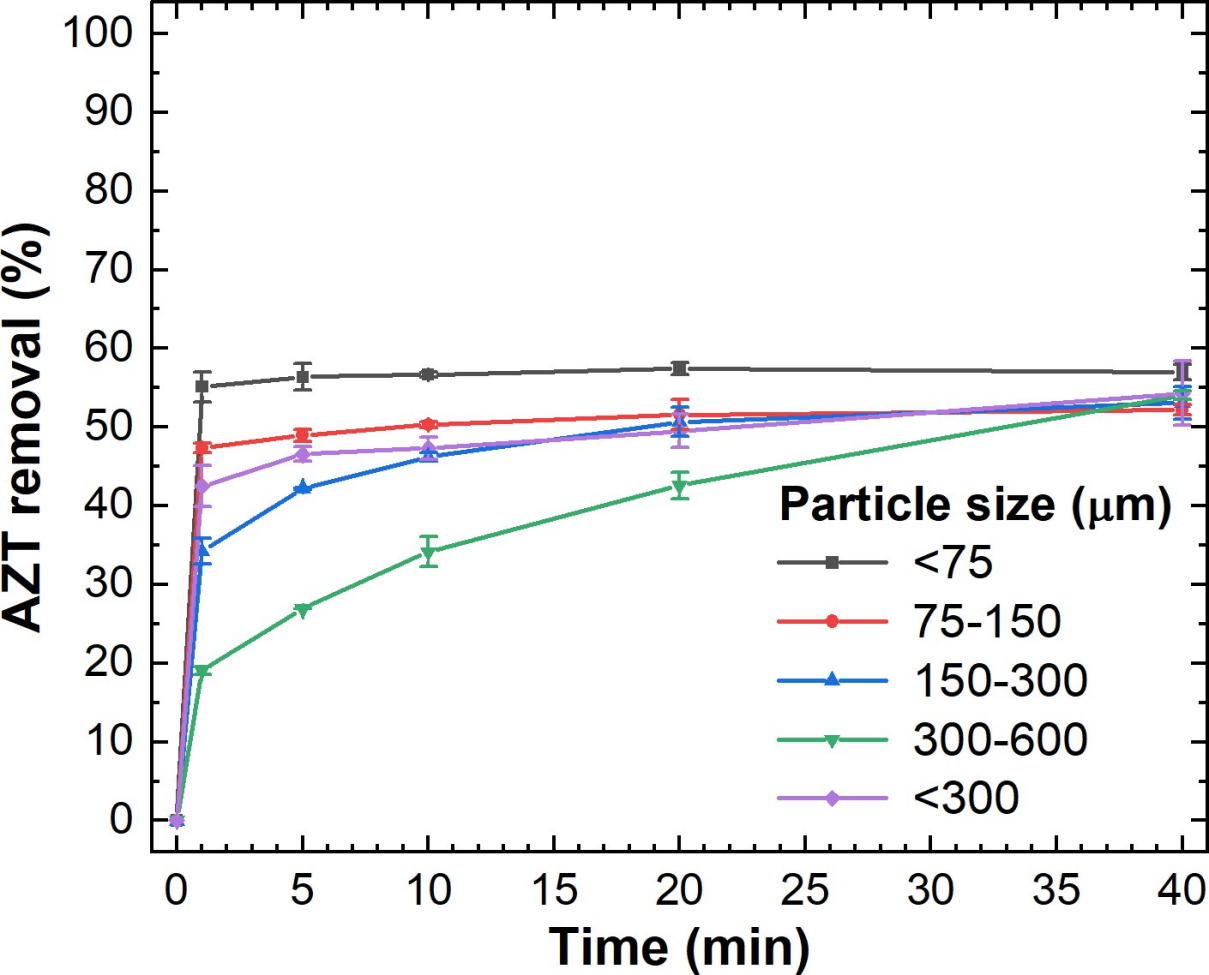
Effect of particle size of L-500 on AZT adsorption. Experimental conditions: C_0_: 100 mg AZT L^-1^, adsorbent dose: 50 g L^-1^, pH: 7.0, T: 20–22°C.

AZT adsorption on L-500 was examined using various sieves, resulting in particle sizes ranging from 75 to 300 μm. The outcomes are illustrated in Fig 6. It is evident that the smallest particle size of L-500 (< 75 μm) results in a higher rate of AZT adsorption, achieving maximum AZT removal (57%) within just 5 minutes of treatment. Smaller particles offer a larger specific surface area, offering more sites for AZT adsorption, thus explaining this phenomenon [70]. Using larger particle sizes of L-500, AZT removal rates approaching 55% were also attained after 40 min of adsorption. Hence, a particle size of < 300 μm has been selected for further experiments.

### Effect of pH on AZT adsorption

**Fig 7:**
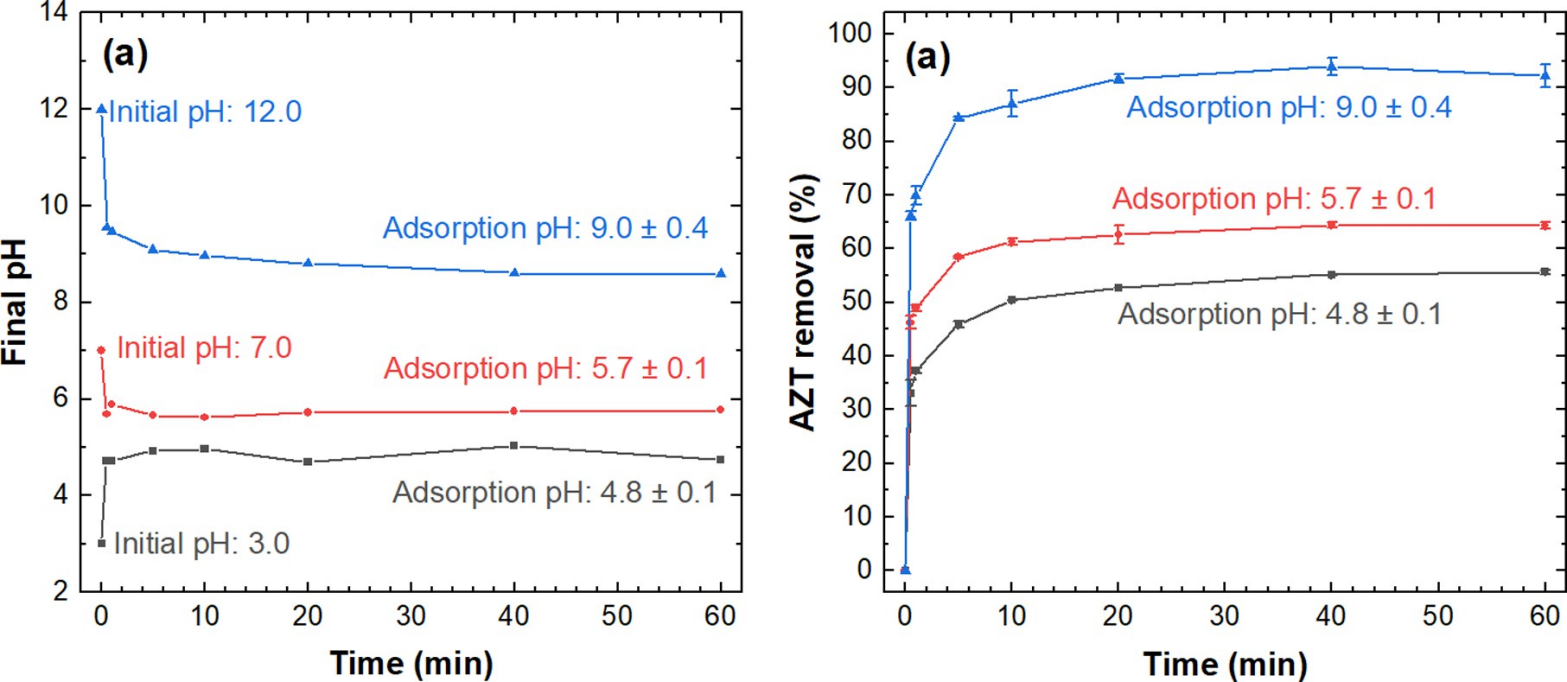
Effect of pH on AZT adsorption using L-500: (a) Final pH, (b) AZT removal. Experimental conditions: C_0_: 100 mg AZT L^-1^, adsorbent dose: 50 g L^-1^, PS: <300 μm, T: 20–22°C.

**Fig 8:**
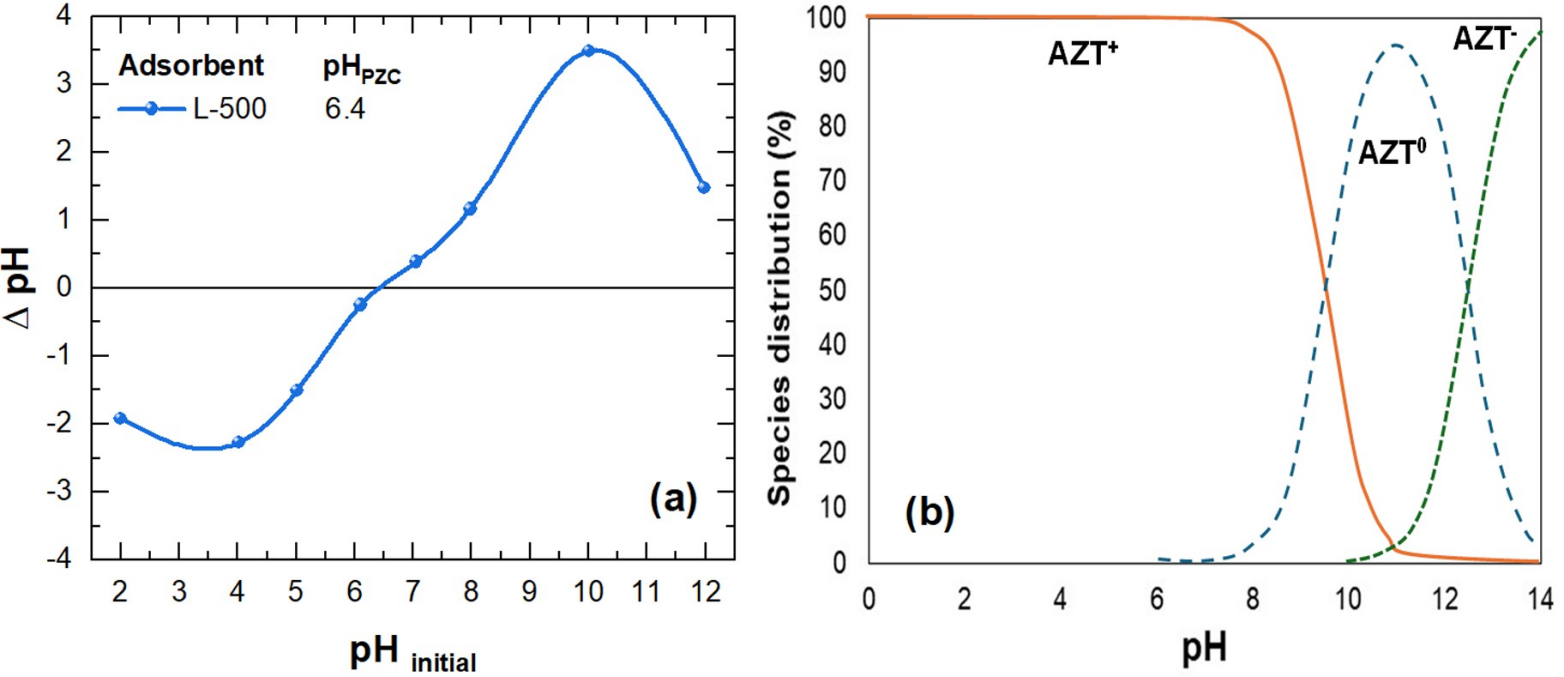
(a) pH_PZC_ of L-100, (b) AZT speciation (Adapted from Rodríguez-López et al. [73]).

Fig 7 shows the effect of pH on AZT adsorption onto L-500 adsorbent. As illustrated in Fig 7A, right after adding the adsorbent (50 g L^-1^) to the AZT solutions, there is a noticeable shift in pH, signifying the strong buffering capacity of L-500. Consequently, the pH levels at which the AZT adsorption process occurs transition from 12.0, 7.0, and 3.0 to 9.0, 5.7, and 4.8, respectively. The results in Fig 7B indicate that AZT removal increases as pH levels rise for all the time intervals considered. At adsorption pH 9.0 near the AZT pKa (~8.7 [71]), the AZT adsorption is maximum, achieving 94% of the AZT removal within a contact time of 40 min. At this pH, AZT adsorption is associated with the electrostatic interaction between the negatively charged surface of the adsorbent L-500, which has a pH_PZC_ of 6.4 (Fig 8A) and the protonated form of AZT (Fig 8B). Additionally, the hydrogen bonding mechanism between the protonated and unionized forms of AZT (Fig 7B) and Si-O^-^ groups present on the L-500 surface can also contribute to AZT adsorption at pH 9.0 [28]. Conversely, AZT removal dropped to 64% and 55% at adsorption pH values of 5.7 and 4.8, respectively. This effect can be attributed to electrostatic repulsion between the predominant cationic moiety of AZT (Fig 8B) and the L-500, which presents a positively charged surface at pH 5.7 (Fig 8A). These electrostatic repulsion forces and their impact on AZT adsorption become more pronounced at lower pH levels, thus elucidating the reduced AZT removal achieved at pH 4.8. The above findings confirm the significant role of pH in the adsorption of AZT onto L-500. Considering that L-500 exhibited relatively high AZT adsorption at an initial pH of 7.0, which falls within the typical range (7.0–9.0) of effluents from municipal wastewater treatment plants [72], pH 7.0 was chosen for further sections.

### AZT adsorption kinetics

The results of the AZT adsorption process onto L-500 were fitted with two widely employed models: the pseudo-first-order (PFO) and pseudo-second-order (PSO) models. S2 and S3 Figs display the fitting of the PFO model (Eq (3)) and PSO models (Eq (4)), respectively. The results of kinetic parameters compiled in Table 4 indicate that both kinetic models fit the experimental data, with R^2^ values exceeding 0.970. However, the PSO kinetic model exhibited superior R^2^ values (0.993–0.999) and low values for APE and Δq, indicating that the qe_calcul_ with this model closely aligns with the qe_exper_. Overall, the interpretation of experimental data is enhanced when employing the PSO kinetic model. These findings suggest that chemisorption is the rate-limiting process in the adsorption of AZT onto L-500 [74], and they indicate a direct relationship between AZT adsorption capacity and the number of active sites present on L-500 (8). Chemical adsorption can encompass hydrogen bonding interactions [28]. Hydrogen bonds could potentially form between the amine group of the AZT and the oxygen atoms present in the functional groups of L-500 [8]. The strong correlation between the adsorption data and PSO kinetics is in line with prior findings on AZT adsorption in water when employing various adsorbents, such as biochar synthesized from rice husk, impregnated with montmorillonite and activated by CO_2_ gas [8], nanocomposites prepared by impregnating activated carbon with metals (Fe, Ag, Zn) [22], natural zeolite (clinoptilolite) modified with surfactants [26], mesoporous silica SBA-15 [24], organoclays [25], Azolla filiculoides-based activated carbon [23] and zeolites [28].

**Table 4:** Kinetic parameters of AZT adsorption using L-500.

C_0_ (mg L^-1^)	qe _exper._ (mg g^-1^)	Pseudo-first order (PFO)					Pseudo-second order (PSO)				
		qe, _calcul_ (mg g^-1^)	k_1_ (min^-1^)	R^2^	APE (%)	Δq (%)	qe, _calcul._ (mg g^-1^)	k_2_ (g mg^-1^ min^-1^)	R^2^	APE (%)	Δq (%)
50	1.054	1.019	1.859	0.991	3.32	4.69	1.051	4.049	0.998	0.28	0.43
60	1.202	1.134	1.365	0.976	5.60	8.05	1.188	2.006	0.996	1.19	2.14
70	1.273	1.200	1.441	0.971	5.70	8.09	1.256	1.989	0.993	1.30	1.89
80	1.261	1.200	1.245	0.974	5.18	7.33	1.262	1.668	0.996	0.07	0.10
90	1.203	1.132	1.246	0.976	5.91	8.36	1.199	1.716	0.996	0.34	0.55
100	1.184	1.125	1.466	0.988	4.92	7.09	1.175	2.382	0.999	0.70	1.27

Experimental conditions: C_0_: 50–100 mg AZT L^-1^, L-500 dose: 50 g L^-1^, pH: 7.0, PS: <300 μm, T: 22°C.

### Adsorption isotherms

**Fig 9:**
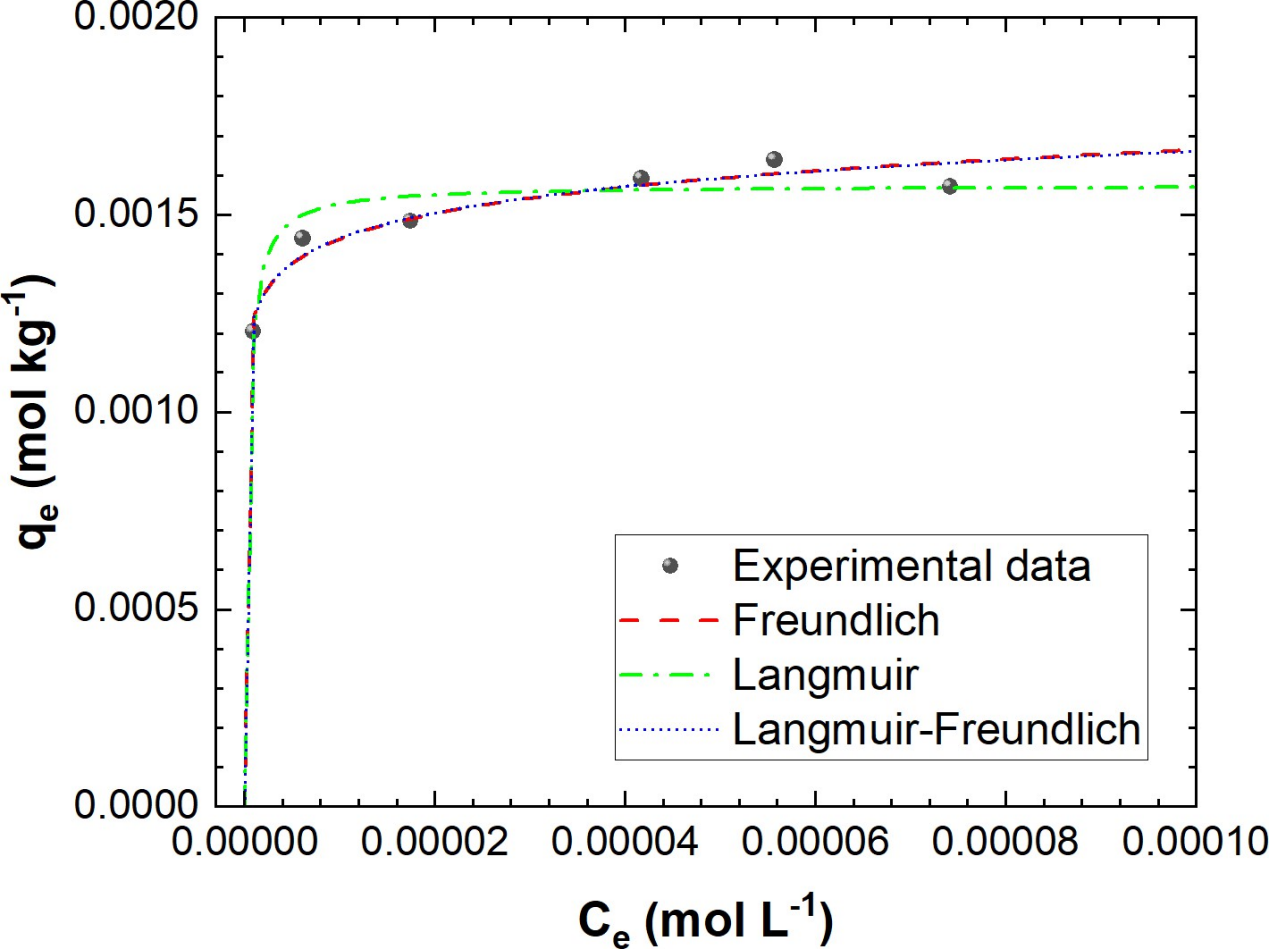
AZT adsorption isotherms. Experimental conditions: C_0_: 90 mg AZT L^-1^ (1.2 x 10^−4^ mol L^-1^), L-500 dose: 5–100 g L^-1^, contact time: 40 min, pH: 7.0, PS: <300 μm, T: 22°C.

Adsorption isotherm models are utilized to investigate the adsorption process for removing pollutants from water, aiming to optimize design parameters [75]. The Langmuir, Freundlich, and Langmuir-Freundlich isothermal models (Table 2) were employed to describe the equilibrium data for AZT adsorption onto L-500. Fig 9 depicts the nonlinear fitting of three models to the experimental data. To accurately calculate the equilibrium constants of the adsorption isotherms, Fig 9 represents Ce and q_e_ in units of mol L^-1^ and mol kg^-1^, respectively, following the suggestion made by Tran et al. [76]. Furthermore, Table 5 summarizes the fitting results (R^2^) and the parameters for each isotherm.

**Table 5:** AZT adsorption isotherms parameters.

Isotherm	Parameter	Value	R^2^	APE (%)	Δq (%)
**Langmuir**	q_m_ (mol kg^-1^)	0.0016	0.89	2.78	4.03
	K_L_ (L mol^-1^)	3322246			
	R_L_	0.0025			
**Freundlich**	K_F_ (mol kg^-1^) (L mol^-1^)^1/n^	0.0030	0.93	2.30	3.06
	1/n	0.063			
**Langmuir—Freundlich**	q_m_ (mol kg^-1^)	0.0058	0.93	2.22	0.06
	Ka (L mol^-1^)	0.1892			
	n_LF_	0.084			

Experimental conditions: C_0_: 90 mg AZT L^-1^ (1.2 x 10^−4^ mol L^-1^), L-500 dose: 5–100 g L^-1^, contact time: 40 min, pH: 7.0, PS: <300 μm, T: 22°C.

Fig 9 demonstrates a strong fit of the experimental data to the three models, as indicated by high correlation coefficients (R²: 0.89–0.93), along with low APE values (2.22–2.78) and Δq values (0.06–4.03), as shown in Table 5. The good agreement between the experimental data and the Langmuir model suggests that, at equilibrium, AZT adsorption corresponds to a homogeneous monolayer on the surface of L-500, with no interactions between the adsorbed species [28]. This model enables the calculation of the maximum adsorption capacity (q_m_) for AZT (0.0016 mol kg^-1^ or 1.20 mg g^−1^), as well as the determination of the separation factor R_L_ (0.0025), which falls within the range of 0 to 1, indicating that the adsorption of AZT onto L-500 is a favorable process [61]. However, we can still observe a strong fit of the experimental data to the Freundlich model (R^2^ = 0.93, APE = 2.30, Δq = 3.06), which considers interactions on multilayer heterogeneous surfaces. These surfaces exhibit varying affinities and interaction energies between their active sites and the adsorbate. This model is instrumental in predicting a favorable AZT adsorption process onto L-500, as indicated by 1/n < 1 [77]. Therefore, there are multiple interactions and adsorption energies between AZT and L-500, involving a combination of adsorption mechanisms. Furthermore, the best fitting to the Langmuir-Freundlich model (R^2^ = 0.93, APE = 2.22, Δq = 0.06) and the heterogeneity index value between 0 and 1 with tends toward zero (n_LF_ = 0.084) indicate that the isotherm approaches a Freundlich-like form and L-500 is a heterogeneous material [78].

As indicated in Table 6, the maximum adsorption capacity (q_m_) achieved for AZT adsorption by L-500 in distilled water (1.20 mg g^−1^) surpasses that of previous reports using materials like lignite (0.395 mg g^−1^) and polyamide nanofibers (0.033 mg g^−1^) [21]. However, other materials, such as biochars [8,21], nanocomposites [22], mesoporous silica [24], nano diatomites [27], and zeolites [28], have reported q_m_ values for AZT adsorption that are superior to those obtained with L-500. This indicates that the adsorbent produced in this study through the thermal activation of WTS (L-500) exhibits a moderate capability for AZT adsorption in aqueous solutions. As a result, its ability to treat wastewater contaminated with AZT will be assessed in the following section.

**Table 6:** Comparison of AZT adsorption capacities in distilled water.

Absorbent	Adsorption capacity (mg g^-1^)	Reference
Nanofiber	0.033	(21)
Lignite	0.395	(21)
Biochar	34.81	(21)
Pristine biochar	33.4	(8)
Carbon dioxide activated biochar-montmorillonite	44.7	(8)
Nanocomposites	14.18	(22)
Mesoporous silica SBA-15	200.5	(24)
Nano diatomites	68–91.8	(27)
Zeolites	7.0–8.5	(28)
WTS calcined at 500°C (L-500)	1.20	This work

### Interaction characterization and reusability

**Fig 10:**
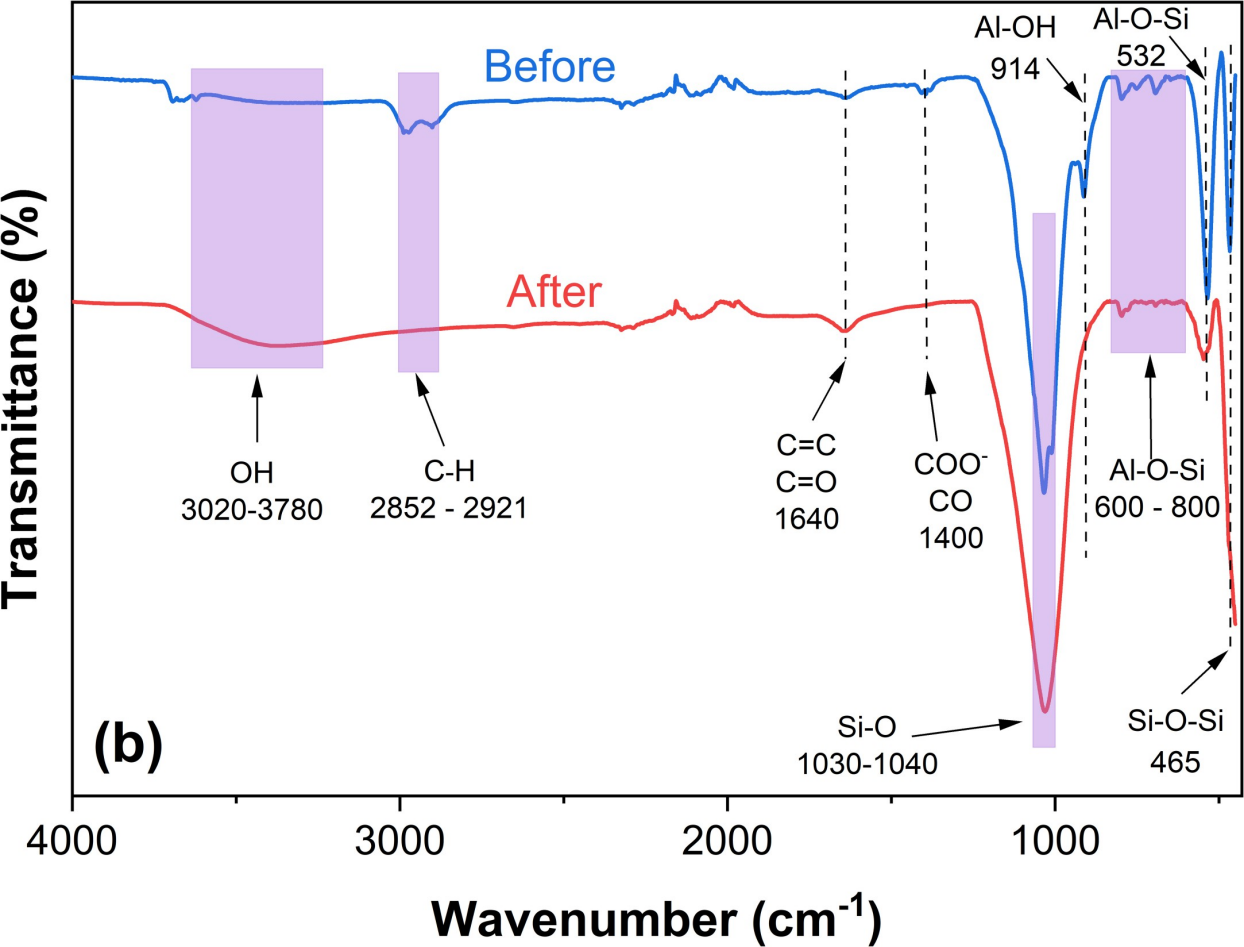
FTIR spectrum of L-500 before and after the adsorption process.

FTIR analysis before and after AZT adsorption on L-500 (Fig 10) was performed to elucidate the molecular interactions between AZT and the material. The spectra reveal significant changes in the material after AZT adsorption.

The shift in the band observed between 3020 and 3780 cm⁻¹ highlights the involvement of the OH functional group from mineral hydroxides in the adsorption process, emphasizing its critical role via hydrogen bonding interactions. Additionally, changes in the signals corresponding to C–H stretching at 2852 and 2921 cm⁻¹ suggest that AZT interacts with these functional groups in the carbonaceous matrix, likely through π-π interactions [79].

More pronounced changes are observed in the bands around 1030 cm⁻¹, 914 cm⁻¹, and 532 cm⁻¹, which are associated with Si–O and Al–OH groups. These findings confirm the vital role of Si–O and Al–O groups, whose negative charge density facilitates interactions with the positively charged nitrogen in AZT during adsorption.

The results indicate that multiple interactions—including π-π stacking, hydrogen bonding, and electrostatic interactions—contribute to the adsorption mechanism.

**Fig 11:**
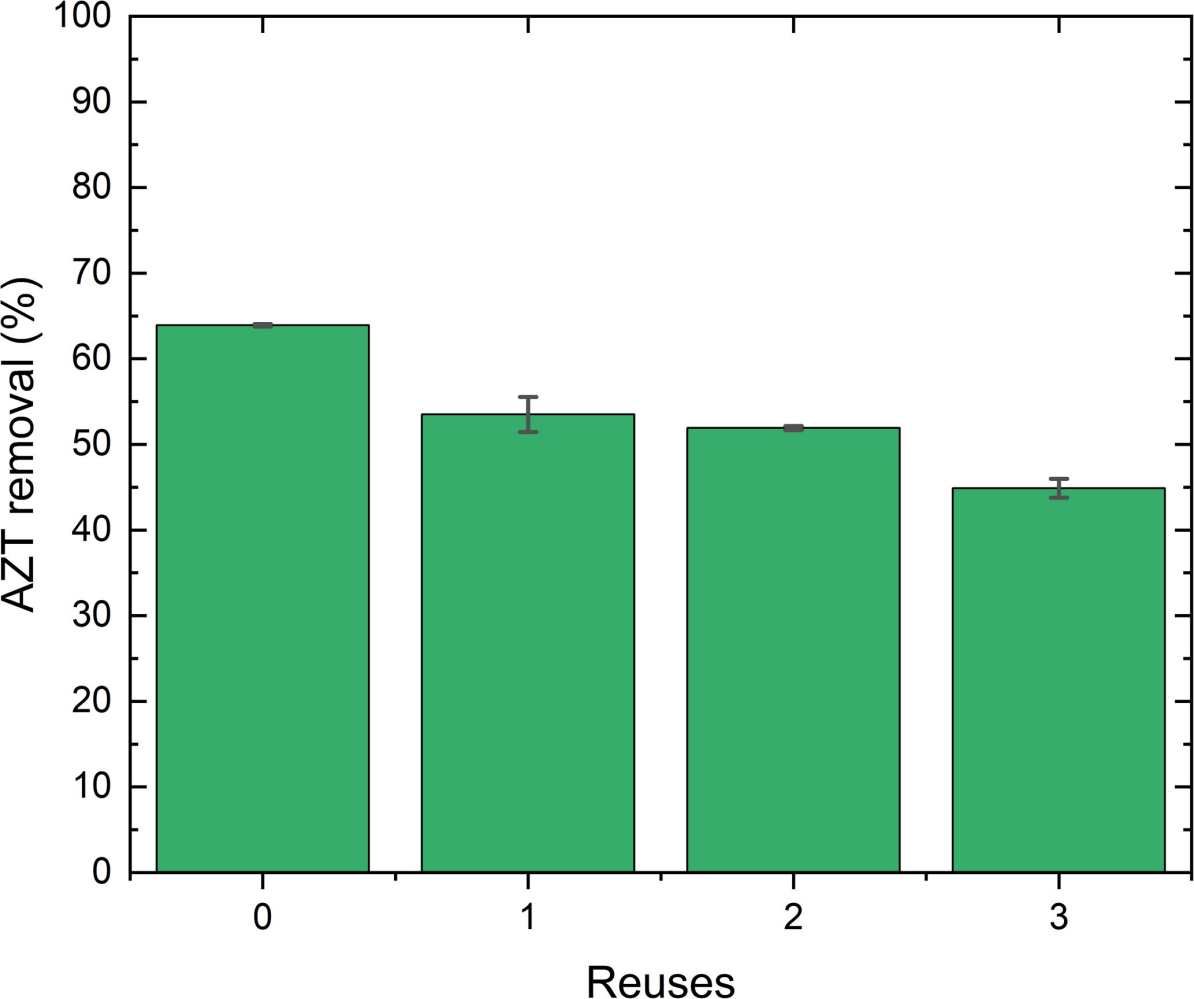
Reuse cycles for L-500 in the AZT adsorption process. Experimental conditions: C_0_: 100 mg AZT L^-1^, L-500 dose: 50 g L^-1^, pH: 7.0, PS: <300 μm, T: 22°C.

Reusability tests are crucial for evaluating the environmental, economic, and industrial feasibility of adsorbents [80,81]. The reusability of the L-500 adsorbent was evaluated over three consecutive cycles (Fig 11). This decrease in the adsorption capacity of L-500 is likely attributed to a loss of reactivity after several reuse cycles [82] and a reduction in available active binding sites on the adsorbent surface during regeneration cycles, particularly since chemisorption was the rate-limiting step in the adsorption process [83].

### AZT adsorption in complex matrices

The adsorption of AZT within complex matrices was evaluated by employing synthetic (S-WW) and real (R-WW) effluents from a municipal wastewater plant, spiked with AZT at a concentration of 100 mg L^-1^. Based on their chemical composition (S1 Table), the tested S-WW is characterized by a high concentration of inorganic salts and presence of organic matter. Similarly, the characterization results for the R-WW sample (S2 Table) reveal high concentrations of anions such as SO₄²⁻ and Cl⁻, along with organic matter (TOC), nitrogen compounds (Total Kjeldahl Nitrogen ‐ TKN, NO₂⁻, NO₃⁻), and phosphorus.

**Fig 12:**
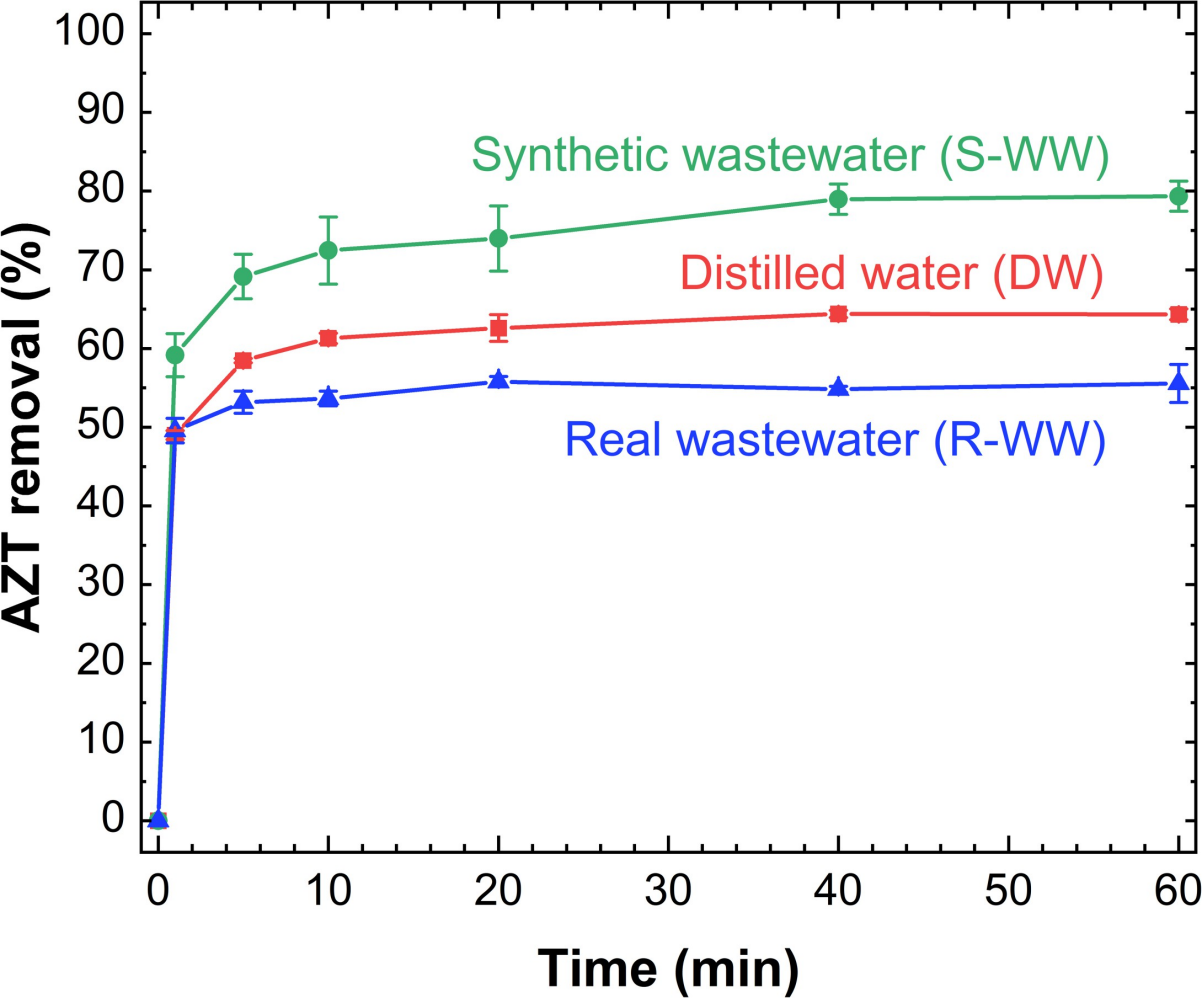
AZT adsorption on L-500 in municipal wastewater effluents. Experimental conditions: C_0_: 100 mg AZT L^-1^, L-500 dose: 50 g L^-1^, pH: 7.0, PS: <300 μm, T: 22°C.

Consequently, cations (Ca^2+^, Mg^2+^, Na^+^, K^+^), anions (SO_4_^2-^, Cl^-^, HCO_3_^-^, HPO_4_^2-^), phosphorus and organic matter can compete with the AZT for the active sites on the adsorbent, affecting the adsorption process in both matrices as reported in previous studies Vrchovecká et al. [21] and De Sousa et al. [28]. As shown in Fig 12, the competitive effect in R-WW is evident, resulting in an 8% reduction in AZT removal after 60 min compared to distilled water (DW). However, AZT adsorption onto L-500 increases by 15% in S-WW compared to DW, achieving approximately 80% removal after 60 minutes of treatment. Additionally, there is a slight increase in the adsorption capacity at equilibrium (1.38 mg g^-1^) in S-WW, as indicated by the results of the PSO kinetic model (S4A Fig). This improvement in adsorption in S-WW matrix can be attributed to the higher pH levels attained in the S-WW during the adsorption process, in contrast to the pH of distilled water (S4B Fig). This elevated pH favors the adsorption of AZT onto L-500. Species such as HCO_3_^-^ and HPO_4_^2-^ contribute to the buffering capacity in S-WW resulting in a smaller decrease in pH during the adsorption process with L-500.

To emphasize the economic benefits of reusing WTS as an AZT adsorbent, Table 7 provides an approximate calculation of L-500 production costs, incorporating factors reported by Kumar et al. [59] and considering the specific conditions of the studied drinking water and wastewater treatment plants. While the values depend heavily on local conditions, L-500, derived from waste generated during the drinking water treatment process, offers a cost-effective solution. Adsorbents produced from waste typically have lower production costs compared to conventional options such as activated carbons, ion exchange resins, and zeolites [84].

**Table 7:** Estimated cost production of L-500.

Adsorbent production factor	Estimated cost (USD kg^-1^)
WTS (precursor)*	0.00
Transportation	0.05
Processing**	0.45
**Total cost**	**0.50**

* WTS is given away by the DWTP.

** Crushing, sieving, calcination and manpower.

## Conclusions and perspectives

In this study, the adsorbent derived from drinking water sludge (WTS) through calcination at 500°C (L-500) demonstrated significant AZT removal efficiency in distilled water. A basic pH condition also enhanced AZT adsorption, primarily due to electrostatic attractions. The PSO adsorption kinetic model indicated that chemisorption is the rate-limiting step in AZT adsorption onto L-500, where the main interactions are π-π stacking, hydrogen bonding, and electrostatic attraction. It was also concluded that the Langmuir-Freundlich equilibrium isotherm model better represented AZT adsorption onto L-500, highlighting the varying interactions and adsorption energies involved in the process. The L-500 was reused for three cycles and showed a reduction of 19% in its AZT removal after the last cycle.

Future research on adsorbents derived from WTS to remove emerging pollutants should focus on continuous column tests, investigate synergistic treatment alternatives, and conduct pilot-scale studies to evaluate practical applications.

Ultimately, this work offers a promising avenue to reuse WTS, addressing two concurrent challenges simultaneously: mitigating water contamination by antibiotics and valorizing waste generated during drinking water treatment. By highlighting the potential of WTS-derived adsorbents, this study contributes to the ongoing efforts to develop sustainable solutions for water treatment and resource recovery.

## Supporting information

S1 Text AZT solutions preparation. (DOCX)

S2 Text AZT concentration measurement. (DOCX)

S3 Text APE and Δq calculation. (DOCX)

S1 Table Composition of synthetic municipal wastewater. (DOCX)

S2 Table Characterization of real municipal wastewater (R-WW). (DOCX)

S1 Fig N_2_ adsorption-desorption isotherm of L-100 and L-500. Inset: Pore size distributions.
(DOCX)

S2 Fig Pseudo-first-order kinetic for AZT adsorption onto L-500: (a) 50 mg L-1, (b) 60 mg L-1, (c) 70 mg L-1, (d) 80 mg L-1, (e) 90 mg L-1, (f) 100 mg L-1. Experimental conditions: C0: 50–100 mg AZT L-1, L-500 dose: 50 g L-1, pH: 7.0, PS: <300 μm, T: 22°C.
(DOCX)

S3 Fig Pseudo-second-order kinetic for AZT adsorption onto L-500: (a) 50 mg L-1, (b) 60 mg L-1, (c) 70 mg L-1, (d) 80 mg L-1, (e) 90 mg L-1, (f) 100 mg L-1. Experimental conditions: C0: 50–100 mg AZT L-1, L-500 dose: 50 g L-1, pH: 7.0, PS: <300 μm, T: 22°C.
(DOCX)

S4 Fig AZT adsorption on L-500 in municipal wastewater effluent. (a) Adsorption capacity, (b) Final pH. Experimental conditions: C0: 100 mg AZT L−1, L-500 dose: 50 g L−1, pH: 7.0, P.S.: <300 μm, T: 22°C.
(DOCX)
