# Supplementary material for: Azithromycin removal from water via adsorption on drinking water sludge-derived materials: Kinetics and isotherms studies
Source: PLoS One. 2025 Jan 9;20(1):e0316487. doi: 10.1371/journal.pone.0316487 (PMC11717256; doi:10.1371/journal.pone.0316487)
Supplement: S1 Text — (DOCX) [file pone.0316487.s001.docx]

**Azithromycin removal from water via adsorption on drinking water sludge-derived materials: kinetics and isotherms studies.**

**S1 Text. AZT solutions preparation.** To prepare the AZT stock solution, 0.1281 g of AZT powder 97.6% purity (Zhejiang Guobang Pharmaceutical Co., Ltd.) was weighed into a 25 mL volumetric flask, and ethanol (C_2_H_6_O) was added to fill to volume to get a concentration of 5000 mg L^−1^ [1].

The AZT solutions used in the adsorption experiments were prepared by withdrawing aliquots from the AZT stock solution in standard volumetric flasks and adjusting the volume with distilled water. For example, to obtain an AZT solution of 50 mg L^−1^, the first 500 µL of AZT stock solution (5000 mg L^−1^) was withdrawn in a 50 mL standard volumetric flask, and then the volume was adjusted with distilled water. The pH of the AZT solution was subsequently set to 7.0 using HCl.

**Reference**

1. Martínez-Polanco MP, Valderrama-Rincón JA, Martínez-Rojas AJ, Luna-Wandurraga, HJ, Díaz-Báez MC, Bustos-López MC, et al. Degradation of high concentrations of azithromycin when present in a high organic content wastewater by using a continuously fed laboratory-scale UASB bioreactor. Chemosphere. 2022; 287: 132191. doi: 10.1016/j.chemosphere.2021.132191
