# Supplementary material for: Azithromycin removal from water via adsorption on drinking water sludge-derived materials: Kinetics and isotherms studies
Source: PLoS One. 2025 Jan 9;20(1):e0316487. doi: 10.1371/journal.pone.0316487 (PMC11717256; doi:10.1371/journal.pone.0316487)
Supplement: S2 Text — (DOCX) [file pone.0316487.s002.docx]

**Azithromycin removal from water via adsorption on drinking water sludge-derived materials: kinetics and isotherms studies.**

**S2 Text. AZT concentration measurement.** Starting from the AZT stock solution, different standard AZT solutions of known concentration in the range of 0 to 100 mg L^−1^ were prepared, following the procedure mentioned in S1 Text. 5 mL of aliquot was withdrawn from each AZT solution, and the volume was adjusted to 10 mL using 5 mL of sulphuric acid 27 N. Then, the solutions were refluxed for 60 min in a thermoblock (Macherey-Nagel Nanocolor Vario 3) at 40°C to get a yellow color solution, and finally, the absorbance was measured at 482 nm using a UV-visible spectrophotometer (Thermo Scientific, Evolution 201) [1, 2, 3]. The calibration curve was elaborated with the data of AZT concentration and absorbance at 482 nm. The AZT concentrations in the samples obtained in adsorption experiments were determined using the calibration curve equation after applying the same procedure applied to the AZT standard solutions.

**References**

1. Davoodi S, Dahrazma B, Goudarzi N, Gorji HG. Adsorptive removal of azithromycin from aqueous solutions using raw and saponin-modified nano diatomite. Water Science and Technology. 2019; 80 (5): 939-949. doi: 10.2166/wst.2019.337
2. Kumar V, Singh SK, Gulati M, Anishetty R, Shunmugaperumal T. Development and validation of a simple and sensitive spectrometric method for estimation of azithromycin dihydrate in tablet dosage forms: application to dissolution studies. Current Pharmaceutical Analysis. 2013; 9 (3): 310-317. doi: 10.2174/1573412911309030009
3. Sultana N, Arayne MS, Hussain F, Fatima A. Degradation studies of azithromycin and its spectrophotometric determination in pharmaceutical dosage forms. Pak J Pharm Sci. 2006 Apr; 19 (2): 98-103. PMID: 16751118
