# Supplementary material for: Azithromycin removal from water via adsorption on drinking water sludge-derived materials: Kinetics and isotherms studies
Source: PLoS One. 2025 Jan 9;20(1):e0316487. doi: 10.1371/journal.pone.0316487 (PMC11717256; doi:10.1371/journal.pone.0316487)
Supplement: S3 Text — (DOCX) [file pone.0316487.s003.docx]

**Azithromycin removal from water via adsorption on drinking water sludge-derived materials: kinetics and isotherms studies.**

**S3 Text. APE and ∆q calculation.** The average percentage error (APE) and the normalized standard deviation (∆q) were calculated using the following equations [1, 2]:

$$APE (\%)=\frac{\sum_{i=1}^{n} \left| \frac{q_{exp}-q_{cal}}{q_{exp}} \right|}{n}\times100 Eq.(SI 1)$$

$$\Delta q(\%)=100\sqrt{\frac{\sum\left( \frac{q_{exp}-q_{cal}}{q_{exp}} \right)^{2}}{n-1}} Eq.(SI 2)$$

where n is the number of data points; q_exp_ and q_cal_ are the experimental and calculated adsorption capacity at equilibrium, respectively.

**References**

1. Paredes-Laverde M, Salamanca M, Silva-Agredo J, Manrique-Losada L, Torres-Palma RA. Selective removal of acetaminophen in urine with activated carbons from rice (Oryza sativa) and coffee (Coffea arabica) husk: Effect of activating agent, activation temperature and analysis of physical-chemical interactions. Journal of Environmental Chemical Engineering. 2019; 7 (5): 103318. doi: 10.1016/j.jece.2019.103318
2. Kumar, N. S., Asif, M., Poulose, A. M., Al-Ghurabi, E. H., Alhamedi, S. S., & Koduru, J. R. Date palm fiber agro-waste biomass for efficient removal of 2, 4, 6-Trichlorophenol from aqueous solution: Characterization, Kinetics, Isotherms studies and Cost-effective analysis. Desalination and Water Treatment. 2024, 100405. doi: 10.1016/j.dwt.2024.100405
