# Supplementary material for: Azithromycin removal from water via adsorption on drinking water sludge-derived materials: Kinetics and isotherms studies
Source: PLoS One. 2025 Jan 9;20(1):e0316487. doi: 10.1371/journal.pone.0316487 (PMC11717256; doi:10.1371/journal.pone.0316487)
Supplement: S1 Table — (DOCX) [file pone.0316487.s004.docx]

**Azithromycin removal from water via adsorption on drinking water sludge-derived materials: kinetics and isotherms studies.**

**S1 Table. Composition of synthetic municipal wastewater (S-WW) [1, 2]**

| **Municipal wastewater** | |
| --- | --- |
| **Compound** | **Concentration (mg L^-1^)** |
| Peptone | 40 |
| Meat extract | 27.5 |
| Urea | 7.5 |
| NaHCO_3_ | 96 |
| KCl | 4 |
| K_2_HPO_4_ | 28 |
| CaSO_4_.2H_2_O | 60 |
| NaCl | 7 |
| CaCl_2_.2H_2_O | 4 |
| MgSO_4_.7H_2_O | 125 |

**References**

1. Paredes-Laverde M, Silva-Agredo J, Torres-Palma RA. Removal of norfloxacin in deionized, municipal water and urine using rice (Oryza sativa) and coffee (Coffea arabica) husk wastes as natural adsorbents. Journal of Environmental Management. 2018; 213: 98-108. doi: 10.1016/j.jenvman.2018.02.047
2. Organization for Economic Co-operation and Development -OECD-. Test No. 303: Simulation Test - Aerobic Sewage Treatment - A: Activated Sludge Units; B: Biofilms. In: OECD Guidelines for the Testing of Chemicals, Section 3. Paris: OECD Publishing; 2001. doi: 10.1787/9789264070424-en.
