# Supplementary material for: Azithromycin removal from water via adsorption on drinking water sludge-derived materials: Kinetics and isotherms studies
Source: PLoS One. 2025 Jan 9;20(1):e0316487. doi: 10.1371/journal.pone.0316487 (PMC11717256; doi:10.1371/journal.pone.0316487)
Supplement: S2 Table — (DOCX) [file pone.0316487.s005.docx]

**Azithromycin removal from water via adsorption on drinking water sludge-derived materials: kinetics and isotherms studies.**

**S2 Table. Characterization of real municipal wastewater (R-WW)**

| **Effluent of municipal wastewater treatment plant** | |
| --- | --- |
| **Parameter** | **Concentration** |
| Total Organic Carbon (TOD) | 16. 448 mg C L^-1^ |
| Total Kjeldahl Nitrogen (TKN) | 41.696 mg N L^-1^ |
| Nitrites (NO_2_^-^) | 0.149 mg NO_2_^-^-N L^-1^ |
| Nitrates (NO_3_^-^) | < 0.226 mg NO_3_^-^-N L^-1^ |
| Total Phosphorus (TP) | 0.766 mg P L^-1^ |
| Chlorides (Cl^-^) | 105.836 mg Cl^-^ L^-1^ |
| Sulphates (SO_4_^-2^) | 75.912 mg SO_4_^-2^ L^-1^ |
