# Supplementary material for: Azithromycin removal from water via adsorption on drinking water sludge-derived materials: Kinetics and isotherms studies
Source: PLoS One. 2025 Jan 9;20(1):e0316487. doi: 10.1371/journal.pone.0316487 (PMC11717256; doi:10.1371/journal.pone.0316487)
Supplement: S1 Fig — Inset: Pore size distributions. (DOCX) [file pone.0316487.s006.docx]

**Azithromycin removal from water via adsorption on drinking water sludge-derived materials: kinetics and isotherms studies.**

**

**S1 Fig. N_2_ adsorption-desorption isotherm of L-100 and L-500.** Inset: Pore size distributions.
