# Supplementary material for: Azithromycin removal from water via adsorption on drinking water sludge-derived materials: Kinetics and isotherms studies
Source: PLoS One. 2025 Jan 9;20(1):e0316487. doi: 10.1371/journal.pone.0316487 (PMC11717256; doi:10.1371/journal.pone.0316487)
Supplement: S2 Fig — Pseudo-first-order kinetic for AZT adsorption onto L-500: (a) 50 mg L-1, (b) 60 mg L-1, (c) 70 mg L-1, (d) 80 mg L-1, (e) 90 mg L-1, (f) 100 mg L-1. Experimental conditions: C0: 50–100 mg AZT L-1, L-500 dose: 50 g L-1, pH: 7.0, PS: <300 μm, T: 22°C. (DOCX) [file pone.0316487.s007.docx]

**Azithromycin removal from water via adsorption on drinking water sludge-derived materials: kinetics and isotherms studies.**

**S2 Fig. Pseudo-first-order kinetic for AZT adsorption onto L-500: (a) 50 mg L^-1^, (b) 60 mg L^-1^, (c) 70 mg L^-1^, (d) 80 mg L^-1^, (e) 90 mg L^-1^, (f) 100 mg L^-1^.** Experimental conditions: C_0_: 50-100 mg AZT L^-1^, L-500 dose: 50 g L^-1^, pH: 7.0, PS: <300 μm, T: 22 °C.
