# Supplementary material for: Azithromycin removal from water via adsorption on drinking water sludge-derived materials: Kinetics and isotherms studies
Source: PLoS One. 2025 Jan 9;20(1):e0316487. doi: 10.1371/journal.pone.0316487 (PMC11717256; doi:10.1371/journal.pone.0316487)
Supplement: S4 Fig — AZT adsorption on L-500 in municipal wastewater effluent. (a) Adsorption capacity, (b) Final pH. Experimental conditions: C0: 100 mg AZT L−1, L-500 dose: 50 g L−1, pH: 7.0, P.S.: <300 μm, T: 22°C. (DOCX) [file pone.0316487.s009.docx]

**Azithromycin removal from water via adsorption on drinking water sludge-derived materials: kinetics and isotherms studies.**

**S4 Fig. AZT adsorption on L-500 in municipal wastewater effluent. (a) Adsorption capacity, (b) Final pH.** Experimental conditions: C_0_: 100 mg AZT L^−1^, L-500 dose: 50 g L^−1^, pH: 7.0, P.S.: <300 µm, T: 22 °C.
